# Draft Genome Sequences of Extended-Spectrum-Beta-Lactamase-Producing *Morganella morganii* Strains AA1 and AV1, Isolated from a Freshwater Lake and *Eicchornia* *crassipes* Roots

**DOI:** 10.1128/genomeA.00527-17

**Published:** 2017-06-15

**Authors:** Pushpa Lata, Subramaniam S. Govindarajan, Feng Qi, Jian-Liang Li, Santosh K. Maurya, Malaya K. Sahoo

**Affiliations:** a Department of Microbiology and Molecular Biology, University of Central Florida, Orlando, Florida, USA; b Analytical Genomics Core, Sanford Burnham Prebys Medical Discovery Institute at Lake Nona, Orlando, Florida, USA; c Applied Bioinformatics Core, Sanford Burnham Prebys Medical Discovery Institute at Lake Nona, Orlando, Florida, USA; d Sanford Burnham Prebys Medical Discovery Institute at Lake Nona, Orlando, Florida, USA; e Department of Pathology, Stanford University School of Medicine, Palo Alto, California, USA

## Abstract

Two strains of *Morganella morganii*, AA1 and AV1, were isolated from freshwater and *Eicchornia crassipes* roots, respectively. Here, we report their draft genome sequences, which are ~3.6 Mb and have 51% G+C content. The predicted coding sequences (3,259 for strain AA1 and 3,345 for strain AV1) encode beta-lactamases, transpeptidases, and penicillin-binding proteins.

## GENOME ANNOUNCEMENT

*Morganella morganii* is a Gram-negative bacterium that belongs to tribe *Proteeae* of the family *Enterobacteriaceae* (1). *M. morganii* is a normal commensal of human gut but is also found in wider environments and has evolved as an opportunistic pathogen (2, 3). In this study, two *Morganella morganii* strains, AA1 and AV1, were isolated from a freshwater lake and the roots of an aquatic weed, *Eicchornia crassipes*, respectively. These two strains exhibit high-level resistance toward beta-lactam antibiotics, ampicillin (>1 mg/ml), and penicillin (0.5 mg/ml). Here, we report the draft genome sequences of these two strains. Genomic DNA was isolated using the QIAamp DNA minikit (Qiagen, Germantown, MD), according to the manufacturer’s instructions. DNA quality was checked using a NanoDrop spectrophotometer (Thermo Scientific) and quantitated by a Qubit 2.0 fluorometer (Life Technologies, Inc.) and Agilent 2100 Bioanalyzer (Agilent Technologies, Santa Clara, CA). The genomic DNA was fragmented and tagged with sequencing adapters in a single-tube enzymatic reaction using the Nextera XT DNA library preparation kit (Illumina, San Diego, CA). The resulting library was sequenced using the Illumina MiSeq sequencing platform (Illumina) and generated 3.8 and 2.7 million reads for strains AA1 and AV1, respectively. The reads were assembled using Minia version 2.0.3, with default parameters and a k-mer size of 121 (4). For *M. morganii* strain AA1, a total of 127 contigs were obtained, the *N*_50_ of the contigs was 119,307 bp, the genome size was 3,567,897 bp, and the G+C content was 51.1%. The *M. morganii* strain AV1 genome comprises a total of 174 contigs, the *N*_50_ of the contigs was 73,996 bp, the genome size was 3,652,729 bp, and the G+C content was 51.2%. The protein-coding regions were predicted by the NCBI Prokaryotic Genome Annotation Pipeline (release 2013 [https://www.ncbi.nlm.nih.gov/genome/annotation_prok/]). Strains AA1 and AV1 contain 3,259 and 3,345 predicted coding sequences in their respective genomes. In the genomes of *M. morganii* strains AA1 and AV1, 97 and 96 RNA genes, respectively, have putative functions assigned on the basis of the annotation. Class C beta-lactamase DHA-1, l,d-transpeptidase, penicillin-binding proteins, and multidrug transporter proteins were annotated in the genome, indicating the beta-lactamase production potential in these strains.

### Accession number(s).

This whole-genome shotgun project has been deposited at DDBJ/ENA/GenBank under accession no. MWUC00000000 and MWUD00000000 for strains AA1 and AV1, respectively. The versions described in this paper are the first versions, MWUC01000000 and MWUD01000000, respectively.
